# Altered Bimanual Kinetic and Kinematic Motor Control Capabilities in Older Women

**DOI:** 10.3390/ijerph20032153

**Published:** 2023-01-25

**Authors:** Joon Ho Lee, Nyeonju Kang

**Affiliations:** 1 Department of Human Movement Science, Incheon National University, Incheon 22012, Republic of Korea; 2 Neuromechanical Rehabilitation Research Laboratory, Incheon National University, Incheon 22012, Republic of Korea; 3 Division of Sport Science, Sport Science Institute, Incheon National University, Incheon 22012, Republic of Korea

**Keywords:** aging, bimanual force control, hand-grip force, motor dexterity

## Abstract

Older women may experience critical neuromuscular impairments interfering with controlling successful bimanual motor actions. Our study aimed to investigate altered bimanual motor performances in older women compared with younger women by focusing on kinetic and kinematic motor properties. Twenty-two older women and 22 younger women performed bimanual kinetic and kinematic motor tasks. To estimate bimanual kinetic functions, we calculated bimanual maximal voluntary contractions (i.e., MVC) and force control capabilities (i.e., mean force, accuracy, variability, and regularity of the total force produced by two hands) during bimanual hand-grip submaximal force control tasks. For bimanual kinematic performances, we assessed the scores of the Purdue Pegboard Test (i.e., PPT) in both hands and assembly tasks, respectively. For the bimanual MVC and PPT, we conducted an independent *t*-test between two groups. The bimanual force control capabilities were analyzed using two-way mixed ANOVAs (Group × Force Level; 2 × 2). Our findings revealed that the older women showed less bimanual MVC (*p* = 0.046) and submaximal force outputs (*p* = 0.036) and greater changes in bimanual force control capabilities as indicated by a greater force variability (*p* = 0.017) and regularity (*p* = 0.014). Further, the older women revealed lower scores of PPT in both the hands condition (*p* < 0.001) and assembly task condition (*p* < 0.001). The additional correlation analyses for the older women showed that lower levels of skeletal muscle mass were related to less bimanual MVC (*r* = 0.591; *p* = 0.004). Furthermore, a higher age was related to lower scores in the bimanual PPT assembly task (*r* = −0.427; *p* = 0.048). These findings suggested that older women experience greater changes in bimanual motor functions compared with younger women.

## 1. Introduction

Well-coordinated bimanual movements in upper extremities requiring neuromuscular integrations contribute to successfully executing the simple activities of daily life (e.g., tying shoelaces and driving vehicles) as well as more complex physical activities (e.g., rowing and climbing). However, older women may experience age-induced changes in bimanual motor functions because of relatively degenerative neuromuscular systems affected by the aging progress combined with changes in sex hormone levels [1]. Prior findings showed a loss of skeletal muscle mass and strength in the bilateral extremities of older women potentially facilitated by decreased estrogen levels after the menopause [2,3]. Moreover, several studies reported that older women revealed altered interhemispheric communications as indicated by excessive inhibitory activations between hemispheres and the atrophy of corpus callosum [4,5]. These findings suggest that older women have functional changes in successfully controlling their bimanual actions.

Previous studies investigated potential changes in hand motor functions in older adults and revealed lesser unilateral force control capabilities in elderly women than elderly men [6,7]. Specifically, Ranganathan and colleagues showed that women showed longer movement times during unimanual dexterity tasks with an increasing age than those for men [6]. Moreover, older women showed higher task errors during multi-finger force control tasks than those in older men [7]. The authors suggested that these changes in unilateral force control capabilities in older women may be associated with hormonal changes such as decreased estrogen levels after the onset of menopause, in addition to aging effects [7]. Focusing on age-induced changes in the hand motor function for women, recent studies have evidenced decreased hand motor functions in older women compared with younger women [8,9]. For example, older women had greater changes in their kinetic and kinematic unilateral force control capabilities, as indicated by a greater force error, force variability, force regularity, and less motor dexterity [9]. In addition, older women showed a greater bilateral deficit phenomenon (i.e., lower force outputs of each limb in a bilateral condition than those in unilateral condition) during a bimanual maximal hand-grip compared with the younger women [8]. Potentially, age-induced changes in unimanual motor functions and a bilateral deficit phenomenon for older women may influence their bimanual motor performances that typically require cooperative motor actions between the hands. Thus, examining the bimanual submaximal motor performances in older women compared with younger women would be a better way to understand and specify changes in bimanual motor patterns in older women. Presumably, this information may provide additional information to develop exercise training protocols specialized for improving bimanual motor functions in older women.

While generating and maintaining isometric forces around a submaximal targeted level using visual information, quantifying the properties of constant force outputs, as referred to the force control capabilities, can be a useful approach for estimating altered bimanual motor performances via visuomotor processing in older women [9,10,11]. Bimanual force control capabilities can be estimated by calculating the accuracy (e.g., root mean square error; RMSE), variability (e.g., coefficient of variation; CV), and regularity (e.g., sample entropy; SampEn) of the total forces produced by two hands [12,13,14]. In particular, SampEn, a temporal structure of variability in non-linear time series force signals, represents motor adaptability (i.e., continuous visuomotor corrections) contributing to successful motor actions. Thus, a higher value of SampEn indicates relatively changeable force control patterns, indicating a greater motor adaptability related to improvements in the bimanual force control capabilities [15,16]. Previous studies reported age-related changes in bimanual force control as indicated by a greater task error, variability, and regularity in older adult groups compared with young controls [11,17]. Furthermore, Lodha and colleagues suggested that a deterioration in bimanual force control performances (e.g., less bimanual force coordination and more asymmetric force production between two hands) for chronic stroke patients was associated with motor impairments in the upper extremity (e.g., lower scores in the Fugl–Meyer assessment and PPT assembly performances) [18,19]. These findings informed that determining altered bimanual force control capabilities in older women may suggest potential changes in bimanual kinetic performances related to sensorimotor processing functions. Although bimanual kinetic and kinematic motor functions at submaximal levels are crucial because the successful activities of daily living are mainly achieved by submaximal motor actions [20,21,22], no one has determined how bimanual submaximal motor functions change in older women [9,11,17,23].

The purpose of this study was to investigate age-induced changes in bimanual motor functions at submaximal levels for older women compared with younger women. For estimating bimanual motor functions, we used (a) MVC and (b) isometric force control tasks at 10% and 40% of MVC (i.e., bimanual kinetic performances) [24,25]. In addition, we applied the Purdue Pegboard Test for assessing the bimanual kinematic performances [26,27,28]. We hypothesized that the older women would show greater aging-induced changes in kinetic and kinematic bimanual motor performances compared with those in younger women.

## 2. Materials and Methods

### 2.1. Participants

Twenty-two older women and 22 younger women voluntarily participated in this cross-sectional two-group design study. The inclusion criteria were healthy participants without musculoskeletal impairments (e.g., sarcopenia) in upper extremities, cognitive impairments, and vision disorders. For the older women group, we included individuals aged 60 years and over who had experienced more than 12 continuous months without menstruation based on the postmenopausal criteria in the literature [29]. For the younger women group, we included individuals aged between 20 and 29 years who reported regular menstrual cycles. To calculate an appropriate sample size, we conducted a priori power analysis based on the pilot data using G*Power software (version 3.1.9.4, Heinrich-Heine-Universität Düsseldorf, Düsseldorf, Germany) and confirmed that 10 participants per group were minimally required in the independent *t*-test and the mixed between-within subjects ANOVA (power > 0.95 and alpha = 0.05). The specific demographic information for the older and younger women groups are shown in Table 1. This study protocol was approved by the Incheon National University’s Institutional Review Board and all participants read and signed an informed consent before starting the experiment.

### 2.2. Experimental Setup

#### 2.2.1. Bimanual Kinetic Performances: MVC and Isometric Force Control Paradigm

**Figure 1:**
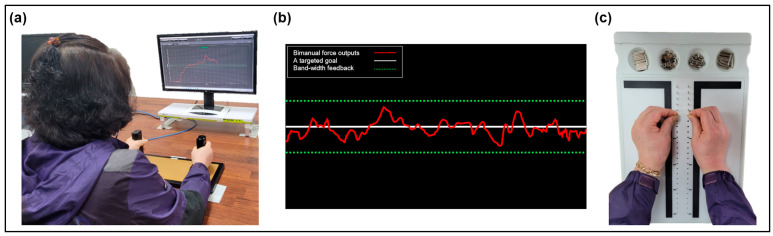
Experimental setup. (**a**) Isometric hand-grip force measurement systems to estimate bimanual MVC and submaximal force control capabilities. (**b**) Three types of visual feedback on the LED monitor. (**c**) Purdue Pegboard Test (PPT).

Consistent with the experimental designs for estimating MVC and force control capabilities [8,25,30], we administered bimanual isometric hand-grip force control tasks. Especially, the MVC value has been widely used for measuring the muscle strength from normal individuals to patients with neuromuscular disorders [31,32,33]. For the tasks, all participants sat 80 cm away from a 54.6 cm LED monitor (1920 × 1080 pixels; a refresh rate = 60 Hz) and put both their arms on the customized platform with comfortable positions (15–20° of shoulder flexion and 20–45° of elbow flexion). Based on recent hand-grip force control studies that examined altered bimanual kinetic functions [8,30], we used a customized isometric hand-grip force measurements system (SEED TECH Co., Ltd., Bucheon, South Korea; Figure 1a) for the experiments. The device includes left and right handles (a diameter = 30 mm) equipped with force transducers (Micro Load Cell-CZL635-3135, range = 220 lbs, Phidgets Inc., Calgary, AB, Canada). While bimanually producing isometric hand-grip forces during the experiments, we instructed participants to fix both their forearms on the customized platform for minimizing any unintentional force outputs produced by other movements of the upper limb joints [8,30].

Participants initially completed two MVC trials (a trial duration = 5 s with 60 s of resting time between trials) while producing bimanual hand-grip forces consistent with previous isometric force control studies [11,25,30]. Further, we selected 10% and 40% of MVC as the targeted force levels because these submaximal force levels show a wide range of forces generated in conducting many activities of daily living [20,21]. Importantly, previous studies showed altered force control strategies depending on these different targeted force levels [12,13,34].

During the bimanual force control tasks at different submaximal force levels, the participants tried to match and sustain bimanual force outputs (i.e., total forces = the sum of forces produced by two hands) around a targeted goal for 20 s. For each trial, we provided two types of visual feedback on the LED monitor, simultaneously (Figure 1b): (a) a red line trajectory = bimanual force outputs and (b) a white line trajectory centered on a screen = a targeted goal. Consistent with prior studies [25,30], additional band-width feedback that potentially contributes to force control improvements was provided. The band-width feedback included upper and lower green lines indicating ±10% of a targeted force level, respectively. All the participants completed 10 consecutive trials for two different targeted force level conditions (i.e., 10% and 40% of MVC). We provided enough resting periods between experimental conditions to minimize the involvement of neuromuscular fatigues on the tasks (e.g., a five minute of resting period after the completion of an MVC task, 30 s of rest between the trials, and 60 s of rest between the submaximal force level conditions) [25,30,35]. Two targeted force level conditions were randomly assigned for each participant.

Using a custom Microsoft Visual C++ Program (Microsoft Corp., Redmond, WA, USA), we administered all the experimental procedures and data collection. All the force data were sampled at the rate of 200 Hz using a 16-bit analog-to-digital converter (A/D; ADS1148 16-Bit 2kSPS and a minimum detectable force = 0.0192 N) and these were amplified by using an INA122 with an excitation voltage of 5 V (Texas Instruments Inc., Dallas, TX, USA). After the data acquisition, we used a custom Matlab Program (Math Works™ Inc., Natick, MA, USA) for further offline analyses.

#### 2.2.2. Bimanual Kinematic Performance: Purdue Pegboard Test

For assessing an individual’s bimanual kinematic function, we applied the Purdue Pegboard Test (PPT; Lafayette Instruments, Lafayette, Indiana) because this test contains a high level of validity and reliability for measuring the bimanual motor dexterity as well as the greater sensitivity of age-related motor changes [36,37,38]. After bimanual force control tasks with at least five minutes of rest, we administered PPT for all participants to minimize task-induced fatigue effects. The PPT includes a testing board with two vertical columns of 25 tiny holes and four cups containing 25 pins at two outsides and 40 washers and 20 collars at the middle two sides on the top of the board (Model 32020A., Lafayette Instrument Company Inc., Lafayette, LA, USA; Figure 1c). Consistent with the guideline manual of the Lafayette Instrument Purdue Pegboard Test based on a previous study [27], the participants comfortably sat right in front of the testing board on the table and were instructed to put (or assemble) as many components as possible in the column from top to bottom. Two bimanual conditions of PPT involved: (a) both hands (the participants simultaneously use both hands while placing pins down both rows for 30 s) and (b) assembly (the participants simultaneously use both hands while assembling pins, washers, and collars for 60 s) for three trials, respectively. We scored the total number of components inserted in the holes for each bimanual condition.

#### 2.2.3. Data Analyses

Initially, all the raw force data were filtered using a bidirectional fourth-order Butterworth filter at 20 Hz of cut-off frequency (Math Works™ Inc., Natick, MA, USA). For 20 s of each trial, we analyzed the middle 16 s of the force signals to minimize the effects of the initial motor corrections and early terminations of the bimanual force outputs. To estimate the bimanual force control capabilities, we calculated four outcome measures: (a) the mean force and (b) force accuracy: RMSE, (c) the force variability: %CV = standard deviation (SD) of the force data/mean force data × 100, and (d) the force regularity: SampEn (Equation (1)) [39,40].

(1) $$\mathrm{SampEn}\left(x,\;m,\;r,\;N\right)=\ln\;\left[\frac{C_{m}\left(r\right)}{C_{m+1}\left(r\right)}\right]$$

which indicates that m is a specific pattern length, r is a criterion of similarity in the time series, and $C_{m}\left(r\right)$ represents the occurrence of repetitive patterns of length m in time series x, indicating force data in the time samples without the self-match [40]. We used a value of m = 2 and r = 0.2 × SD of the force data corresponding to a previous study [39].

#### 2.2.4. Statistical Analyses

The bimanual force control dependent variables (i.e., mean force, RMSE, CV, and SampEn) were analyzed using two-way mixed measure ANOVAs (Group × Force Level; 2 × 2) with repeated measures on the last factor. For the post hoc analysis, we used Bonferroni’s pairwise comparisons. Moreover, the independent *t*-test was used for comparing the PPT scores, bimanual MVC, age, and body composition variables (i.e., the weight, skeletal muscle mass, and body fat mass) between the younger women and older women groups. Given that the violation of the normality assumption was observed for the body mass index (BMI), we conducted the Mann–Whitney U test. In addition, for the older women group, we performed Pearson’s correlation analyses to identify potential relationships between age, the three body composition variables, and bimanual motor functions (i.e., kinetic and kinematic variables). For estimating the potential relation of BMI to other variables, we used Spearman’s correlation analysis. All the statistical analyses were conducted using IBM SPSS Statistics 22 (SPSS Inc., Chicago, IL, USA) and the alpha levels were set at 0.05 for the statistical tests.

## 3. Results

### 3.1. Bimanual MVC and Submaximal Isometric Force Generation

**Figure 2:**
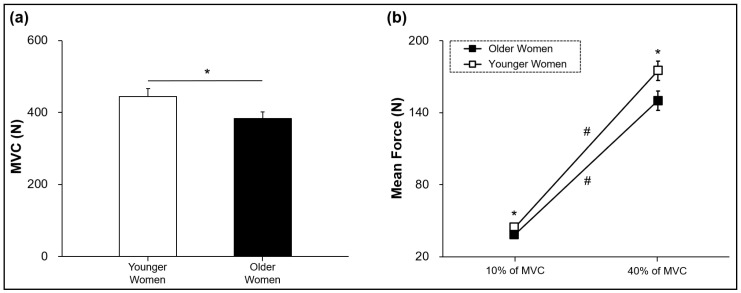
Bimanual maximal and submaximal isometric forces (M ± SE). (**a**) Maximal force showing a significant group main effect. (**b**) Submaximal mean force showing a significant Group × Force Level interaction. Asterisk (*) indicates a significant difference between two groups. Number sign (#) denotes a significant difference between 10% and 40% of MVC.

To confirm potential differences in the bimanual muscle strength between the two groups, we conducted independent *t*-tests on the bimanual MVC values. The analyses indicated that the values of bimanual MVC were significantly lower in the older women group compared with the younger women group (*t*_42_ = −2.059 and *p* = 0.046; Figure 2a). Furthermore, a two-way mixed ANOVA on the mean force showed a significant Group × Force Level Condition (2 × 2) interaction [*F* (1, 42) = 4.707; *p* = 0.036; partial η^2^ = 0.101; Figure 2b]. The post hoc analysis revealed that the mean force during the submaximal bimanual force generation in the older women group was significantly lower across all the targeted force levels (*p* = 0.039 at 10% of MVC and *p* = 0.037 at 40% of MVC) than the younger women group, and further the mean force significantly decreased from 40% to 10% of the MVC for both groups (*p* < 0.001; Figure 2b). These findings indicate that while bimanually executing isometric contractions, the older women showed less muscle strength as well as less submaximal force generation around the targeted force lines than those in the younger women.

### 3.2. Bimanual Kinetic Performances: Force Accuracy, Variability, and Regularity

**Figure 3:**
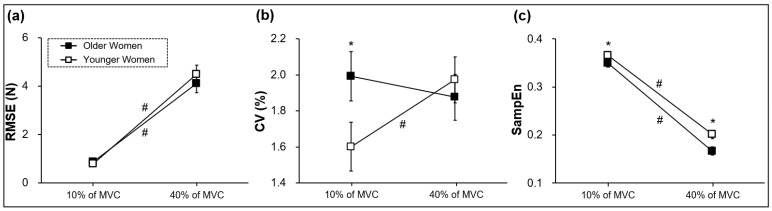
Bimanual force control performances (M ± SE). (**a**) Force accuracy showing a significant Force Level main effect. (**b**) Force variability showing a significant Group × Force Level interaction. (**c**) Force regularity showing significant Group and Force Level main effects. Asterisk (*) indicates a significant difference between two groups. Number sign (#) denotes a significant difference between 10% and 40% of MVC.

The Group × Force Level Condition (2 × 2) mixed ANOVA on the RMSE showed a significant Force Level main effect [*F* (1, 42) = 182.035; *p* < 0.001; partial η^2^ = 0.813; Figure 3a]. Collapsed across two different groups, the RMSE was significantly lower at 10% of MVC than 40% of MVC. Despite less maximal and submaximal bimanual force generation for the older women group (Figure 2), their RMSE was not significantly different compared with the younger women group.

The analysis on the CV showed a significant Group × Force Level Condition (2 × 2) interaction [*F* (1, 42) = 6.136; *p* = 0.017; partial η^2^ = 0.127; Figure 3b]. The follow-up tests revealed that the older women group produced a higher CV than the younger women group at 10% of MVC (*p* = 0.048), whereas no significant difference in the CV between the groups appeared at 40% of MVC (*p* = 0.597). The younger women group significantly reduced the CV from 40% to 10% of MVC (*p* = 0.011), whereas this change was not observed in the older women group (*p* = 0.407). These findings demonstrate that the older women produced more force variability in comparison to the younger women at the lower targeted force level, and they failed to decrease the force variability from a higher to lower submaximal force level.

The two-way mixed ANOVA on the SampEn found two significant main effects: (a) Group: *F* (1, 42) = 6.532; *p* = 0.014; partial η^2^ = 0.135 and (b) Force Level: *F* (1, 42) = 893.943; *p* < 0.001; partial η^2^ = 0.955; Figure 3c. Collapsed across two targeted force levels, the SampEn in the older women group was significantly lower than the younger women group. These findings indicate that the older women produced a greater force regularity while bimanually executing isometric force control tasks than the younger women.

### 3.3. Bimanual Kinematic Performances: Both Hands and Assembly Tasks in PPT

**Figure 4:**
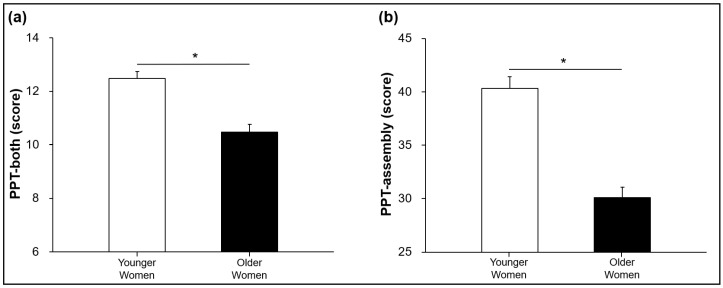
Purdue Pegboard Test scores in both hands and assembly tasks (M ± SE). (**a**) PPT scores in both hands task showing a significant difference between groups. (**b**) PPT scores in bimanual assembly task showing a significant difference between groups. Asterisk (*) indicates a significant difference between two groups.

The independent *t*-tests showed that the number of components successfully inserted into the holes for the older women group was significantly less than those in the younger women group across two PPT task conditions: (a) both hands condition (*t*_42_ = −5.101 and *p* < 0.001; Figure 4a) and (b) assembly condition (*t*_42_ = −6.928 and *p* < 0.001; Figure 4b). These findings indicate that older women showed significant aging-induced changes in the bimanual motor dexterity compared to the younger women.

### 3.4. Correlation Findings: Age, Body Composition Variables, and Bimanual Motor Functions

**Figure 5:**
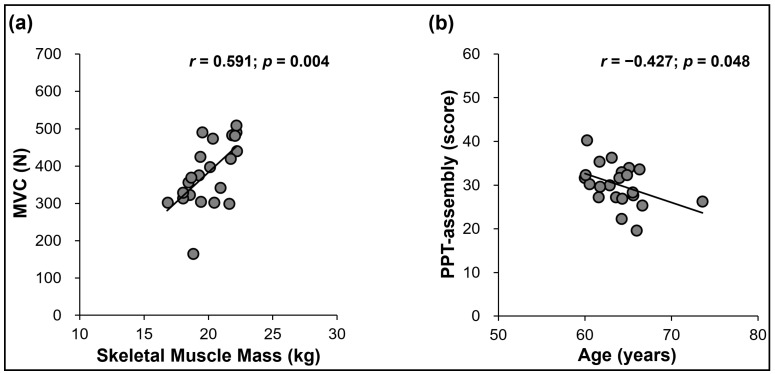
Correlation findings between skeletal muscle mass versus bimanual MVC and age versus PPT scores in bimanual assembly task for the older women group. (**a**) Lower levels of skeletal muscle mass were significantly related to less bimanual maximal forces. (**b**) Higher age was significantly related to lower bimanual PPT assembly scores.

The primary analyses identified significant differences between the groups for age, the body composition variables (i.e., skeletal muscle mass, body fat mass, and BMI), and the bimanual kinetic and kinematic variables (i.e., MVC, mean force, SampEn, CV, and PPT scores in both hands and assembly task conditions). Thus, we conducted correlation analyses between the age, body composition, and bimanual motor function variables for the older women group (Table 2). The analyses revealed that lower levels of skeletal muscle mass were significantly related to lower bimanual MVC levels (Figure 5a). In addition, a higher age was significantly related to lower bimanual PPT assembly scores (Figure 5b).

## 4. Discussion

This study investigated the changes in bimanual motor functions in older women by estimating bimanual kinetic (maximal and submaximal isometric force generation and force control capabilities) and kinematic (i.e., hand motor dexterity) performances. The older women showed significant aging-induced changes in bimanual kinetic performances, as indicated by the lower bimanual MVC and mean submaximal force, higher force variability, and increased force regularity compared with those in younger women. Moreover, the older women group showed significantly less PPT scores for the assembly and both hands task conditions than the younger women group. For the older women, we found significant correlations between a decrease in skeletal muscle mass and less bimanual MVC and between an increased age and lower scores in the bimanual PPT assembly task.

Beyond the muscle weakness patterns observed in unilateral hand conditions [41,42,43], our findings confirmed that the older women had less bimanual hand-grip muscle strength (i.e., less bimanual MVC) than the younger women. Recent studies suggested that older women may experience more aging-induced changes in physical functions, such as decreased muscle strength and power [2,9,44]. The authors posited that these changes may be related to a potential dysregulation in the muscle protein turnover (i.e., an imbalance between the synthesis and degradation of cellular protein) induced by the decreased expression of the insulin-like growth factor (IGF)-1, increased skeletal muscle apoptosis causing muscle atrophy, and deteriorated coupling between myosin and actin fiber [45,46,47]. Recently, a behavioral study revealed that the declines in hand-grip strength caused by the loss of skeletal muscle mass appeared in the bilateral upper limbs in older women [48]. Presumably, the loss of bimanual hand-grip muscular strength in older women may be associated with the effect of the aging process normally facilitating progressive neuromuscular deteriorations in the peripheral systems (e.g., a loss of motor units and neuromuscular junctions and reduced activation in the motor neuron pools) [49,50,51].

Despite the fact that less submaximal bimanual forces were produced by the older women than the younger women, insignificant differences in the force accuracy between the two groups raised a possibility of a relatively lower task accuracy in the older women because the force error proportionally decreases with less force outputs [52,53]. On the other hand, it is possible that the bandwidth feedback (i.e., ±10% of a targeted force level) used for this study may be beneficial for online bimanual motor corrections in older women. Recent findings revealed that the presence of online-bandwidth visual feedback during unilateral force control tasks influenced the task accuracy when the performers attempted to complete more difficult task requirements (e.g., force control with non-dominant hand at a higher targeted force level) [25]. Potentially, providing additional bandwidth visual feedback during bimanual force control tasks may be advantageous for older women who presumably have an impaired visuomotor processing function compared to younger women.

The greater force variability and higher force regularity patterns in older women during bimanual submaximal force control tasks expanded the recent findings that revealed greater changes in the unimanual force control as well as more bilateral deficit phenomenon in older women than younger women [8,9]. To the best of our knowledge, our findings are the first to report aging-induced changes in bimanual force control capabilities at submaximal targeted force levels for older women compared with younger women. When participants execute bimanual force control tasks, motor variability can be estimated by two approaches: (a) the linear time-series of the force outputs (e.g., a greater CV of forces indicating more force variability related to the noise of motor outputs) and (b) the nonlinear temporal structures of the force outputs (e.g., lower values of SampEn showing more force regularity related to more stereotyped motor actions). Thus, the increased variability and regularity of the force outputs denotes the aging-induced changes in the force control performances while processing simultaneous external visual information [12,54,55,56]. Taken together, our bimanual force control findings raised a possibility of changes in the sensorimotor processing functions of older women.

Moreover, bimanual force control changes in older women commonly observed at the lower targeted force level (i.e., 10% of MVC) support previous findings that the elderly population and patients with neurological diseases showed altered bimanual force control capabilities at lower targeted force levels [10,54]. Focusing on aging-related changes in bimanual motor functions, previous studies revealed that older adults, compared with younger adults, showed aging-related changes at a different task difficulty and symmetry conditions during bimanual motor tasks [23,57]. Specifically, older adults indicated higher values of error and variability while conducting bimanual hand-grip force tracking at both the symmetrical in-phase condition and alternating anti-phase task condition [57]. Rudisch and colleagues reported aging-induced changes by showing a reduction in the sustained time on a target (i.e., increased deviations to a target line) and reduced bimanual coupling at in- and anti-phase task conditions [23]. Moreover, for older adults, a less complex structure of force (i.e., decreased motor adaptability) appeared at an alternating task condition [23]. The potential mechanisms underlying aging-induced changes in the bimanual force control capabilities in older women may involve the changes in interhemispheric communications because of aging effects as well as reduced sex hormonal levels after menopause. Previous studies evidenced that a greater interference of interhemispheric inhibitory activations and altered activations in the corpus callosum appeared in older women with relatively lower estrogen levels [4,5,58]. Moreover, aging-related physiological noise caused by higher oscillations of common synaptic inputs to motor neurons may increase the force variability and the larger size of motor units (i.e., more muscle fibers controlled by an alpha motor neuron) may interfere with adaptive muscle contractions, which potentially increased the force regularity for the older women [15,59,60].

In addition to the altered bimanual kinetic performances, we found that older women revealed decreased bimanual kinematic performances as indicated by lower motor dexterity scores in the PPT. Aging-related changes in motor inhibitory systems in cortical and subcortical regions may be one possible cause of deteriorating bimanual kinematic motor performances for older women in addition to dysfunctional peripheral neuromuscular systems. Previous studies confirmed that the γ-aminobutyric acid (GABA) levels tended to decrease in older adults [61,62] and these degeneration patterns in GABA levels increased in older women [63]. Importantly, the impaired integrity of the GABA neurotransmitter system appears to influence the bimanual dexterity and motor control functions through dysregulations of the appropriate motor responses (e.g., adaptive motor actions) [64,65]. Furthermore, a recent neuroimaging study reported that early perimenopausal women showed a long silent period assessed by transcranial magnetic stimulation (TMS), indicating better GABAergic-mediated inhibitory system than those in late perimenopausal women [66,67]. Perhaps, these findings support the proposition that aging-induced changes in bimanual kinetic and kinematic performances in older women may be influenced by changes in the central and peripheral neuromuscular systems.

A previous study reported that a decrease in the skeletal muscle mass was significantly associated with less unimanual hand-grip strength for the older women [68]. Our correlation findings expanded these results by showing that less skeletal muscle mass was related to reduced bimanual hand-grip strength in the older women. These findings indicated that the loss of skeletal muscle mass in older women may be a critical indicator interfering with bimanual muscle strength. Moreover, we found that increased age in older women was significantly related to lower scores in the bimanual assembly task, which is consistent with prior findings [37,69]. In 10-year segments (60–69 years, 70–79 years, and over 80 years), the older women showed significant decreases in unimanual and bimanual dexterity scores with increasing age [37]. Murata and colleagues showed progressive reductions in PPT scores and decreased tactile sensitivity for older women with an increasing age [69]. Presumably, older women may experience aging-induced progressive reductions in bimanual dexterity affected by altered sensorimotor processing capabilities.

Despite decreased bimanual kinetic and kinematic performances for the older women in this study, these findings should be carefully interpreted. First, this study focused on older women with a mean age less than 70 years old (means ± SD age = 63.9 ± 3.0). Given the possibility that aging-related neuromuscular changes may be additionally affected by different ranges of age in older groups (e.g., young–old aged 60 to 75 and older–older aged 75 to 85) [37,48,69,70], future studies need to compare the altered bimanual motor functions between these older women groups. Moreover, the current study did not directly examine the endogenous changes in hormonal levels and altered central and peripheral neuromuscular systems that can be measured by TMS and electromyography techniques. Given that several findings suggested that impaired hand motor functions were potentially related to altered estrogen levels, GABA levels, and involvements of the motor unit pools in older women [65,66,71,72], future studies should investigate potential relationship between altered bimanual motor functions and hormonal levels and neuromuscular systems in older women.

## 5. Conclusions

The current study revealed that the older women showed aging-induced changes in bimanual motor performances compared with the younger women. We found a significant reduction in both bimanual kinetic and kinematic performances in the older women, as indicated by weaker maximal and submaximal forces, greater changes in bimanual force control capabilities (i.e., increased force variability and regularity), and lower bimanual motor dexterity scores. Our correlation findings showed that having less skeletal muscle mass was related to weaker bimanual maximal forces for older women, and increased age was related to lower bimanual motor dexterity. These findings suggest that older women may experience aging-induced changes in bimanual kinetic and kinematic motor control capabilities at submaximal levels.

## Figures and Tables

**Table 1:** Demographic and clinical information for participants.

Characteristics	Younger Women Group	Older Women Group	*p*-Value
Sample size	22	22	-
Age (years)	22.3 ± 1.8	63.9 ± 3.0	<0.001 **
Time since menopause (years)	-	14.0 ± 6.5	-
Handedness (left:right)	1:21	0:22	-
Weight (kg)	55.2 ± 6.7	57.6 ± 6.4	0.210
Skeletal muscle mass (kg)	22.0 ± 2.4	20.0 ± 1.6	0.003 **
Body fat mass (kg)	14.8 ± 4.4	20.5 ± 5.1	<0.001 **
BMI (kg/m^2^)	21.0 ± 2.4	23.8 ± 2.6	0.046 *

Data are means ± SD. Abbreviation. BMI: body mass index. * indicates *p* < 0.05. ** indicates *p* < 0.01.

**Table 2:** Correlation findings between age, body composition, and bimanual motor functions.

Variables	MVC	Mean Force		SampEn		CV		PPT	
		10%	40%	10%	40%	10%	40%	Both Hands	Assembly
Age	−0.092 (0.683)	−0.093 (0.682)	−0.107 (0.635)	0.014 (0.951)	0.150 (0.506)	−0.049 (0.828)	−0.326 (0.139)	−0.287 (0.196)	−0.427 * (0.048)
Skeletal muscle mass	0.591 ** (0.004)	0.580 ** (0.005)	0.578 ** (0.005)	−0.221 (0.324)	−0.025 (0.911)	−0.170 (0.450)	−0.049 (0.828)	−0.289 (0.192)	−0.016 (0.944)
Body fat mass	0.033 (0.979)	0.006 (0.983)	0.005 (0.881)	0.034 (0.378)	−0.198 (0.378)	0.034 (0.882)	0.224 (0.315)	−0.192 (0.393)	−0.051 (0.822)
BMI	0.043 (0.848)	0.015 (0.948)	0.022 (0.922)	−0.004 (0.984)	−0.385 (0.077)	0.129 (0.567)	0.412 (0.057)	−0.159 (0.480)	−0.026 (0.908)

Data are Pearson’s correlation coefficient (*r*) and *p*-value. Abbreviation. BMI: body mass index; CV: coefficient of variation; MVC: maximal voluntary contraction; PPT: Purdue Pegboard Test; SampEn: sample entropy. * indicates *p* < 0.05. ** indicates *p* < 0.01.

## Data Availability

The data are available upon requested from corresponding author.
